# Molecular Identification of *Fonsecaea monophora*, Novel Agent of Fungal Brain Abscess

**DOI:** 10.3201/eid3006.240077

**Published:** 2024-06

**Authors:** Sudesh Gourav, Gagandeep Singh, Mragnayani Pandey, Bhaskar Rana, Sonakshi Gupta, Himanshu Mishra, Immaculata Xess

**Affiliations:** All India Institute of Medical Sciences, New Delhi, India

**Keywords:** *Fonsecaea monophora*, fungal brain abscess, disseminated phaeohyphomycosis, fungi, India

## Abstract

A 3-year-old patient in India experiencing headaches and seizures was diagnosed with a fungal infection, initially misidentified as *Cladophialophora bantiana*. Follow-up sequencing identified the isolate to be *Fonsecaea monophora* fungus. This case demonstrates the use of molecular methods for the correct identification of *F. monophora*, an agent of fungal brain abscess.

A 3-year-old boy was admitted to the All India Institute of Medical Sciences, New Delhi, India, with headache for 3 weeks and 2 episodes of seizures. He had no history of fever, vomiting, or altered senses; no history suggestive of tuberculosis; and no predisposing conditions. Fundoscopic examination revealed bilateral papilledema. Magnetic resonance imaging of the brain showed multiple contrast enhancing lesions. Contrast enhanced computed tomography of chest and abdomen revealed well-defined nodules in the right lung and both lobes of the liver. Cerebrospinal fluid examination showed a glucose level of 51 mg/dL (reference range 40–70 mg/dL), protein level of 82 mg/dL (reference range 12–60 mg/dL), and leukocyte count of 45 cells/mm^3^ (reference range 0–20 cells/mm^3^) with 22% neutrophils. We found persistent eosinophilia (up to 31%) on sequential blood counts.

We conducted a potassium hydroxide-calcofluor white examination of the ultrasound-guided liver biopsy sample, which showed dematiaceous septate hyphae 3–6 μm in diameter. The hyphae showed bulbous dilatations at irregular intervals. Light microscopic examination also showed dematiaceous septate hyphae. A brain biopsy taken from the right parietal lesion showed similar dematiaceous septate hyphae on potassium hydroxide-calcofluor white examination. Histologic examination of both samples showed granulomatous inflammation with hyphae of dematiaceous fungi. We administered intravenous liposomal amphotericin B and voriconazole and continued treatment for 9 weeks.

We performed a fungal culture of the liver biopsy, but no growth was detected. The brain biopsy sample grew dark brown-black velvety colonies on sabouraud dextrose agar at both 25° and 37°C after 9 days of incubation. Lactophenol cotton blue mount showed septate brown hyphae with moderately long sparsely branched chains of smooth oval brown conidia (Figure 1). The organism was initially misidentified as *Cladophialophora bantiana* because of its known preponderance in cases of invasive fungal disease of the central nervous system (CNS).

**Figure 1:**
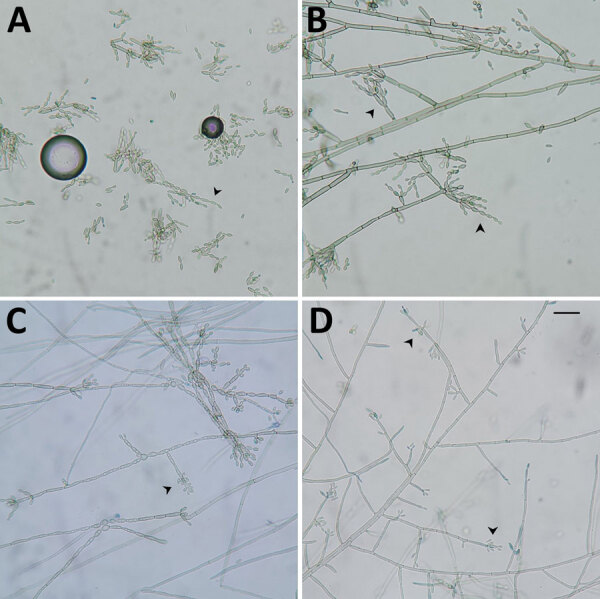
Lactophenol cotton blue mount of a culture from a brain biopsy sample from a 3-year-old patient in India with *Fonsecaea monophora* infection, showing dematiaceous septate hyphae with different types of conidiation (arrowheads). A) Multicelled sessile conidial chains resembling genus *Cladophialophora*, leading to the initial misidentification. B) *Fonsecaea*-type conidiation. C) *Rhinocladiella*-type conidiation. D) Asterisks of *Fonsecaea*-type conidiation.

We repeated brain imaging after the intravenous treatment course. Imaging showed the lesions had greatly reduced in size and number. We started the patient on an oral combination of flucytosine and voriconazole. After 8 weeks on the oral regimen, the patient’s symptoms resolved, and imaging showed near-complete radiologic resolution of the lesions. We stopped antifungal treatment. We followed up with the patient after a year, and there was no recurrence.

We retrospectively performed molecular identification of the culture isolate from the brain biopsy sample. We ran conventional PCR on the extracted DNA targeting the internal transcribed spacer (ITS) region of the 18s rDNA. We conducted sequencing by using an ABI 3730XL automated sequencer (ThermoFisher Scientific, https://www.thermofisher.com).

We edited sequences by using Geneious Prime 2023.2.1 (Geneious, https://www.geneious.com). We conducted a basic local alignment search tool inquiry of the sequenced region in GenBank, resulting in 98.95% identity with *F. monophora* fungal strain CBS 269.37. We analyzed phylogenetically informative polymorphic sites in the ITS region of *Fonsecaea* spp. according to de Hoog et al. (*1*) and found 12 of 13 bases were identical with *F. monophora*, confirming the identification (GenBank accession no. OR773059) (Table).

**Table:** Phylogenetically informative polymorphic sites in the ITS region of *Fonsecaea* spp., showing 12 of 13 bases from the isolate cultured from the brain biopsy of a 3-year-old patient were identical with *F. monophora* fungus*

Species	ITS 1 (206)									5.8s (169)		ITS 2 (152)			
	15	41	47	79	99	100	109	113		46		47	119	121	144
*F. pedrosoi*	C	C	A	T	C	T	T	A		C		G	T	T	T
*F. monophora*	T/–†	T	T	G	T	C	C	G		T		A	A	C	C
Study isolate	T	T	T	G	T	C	C	G		C		A	A	C	C

*ITS, internal transcribed spacer. †Presence of thymine or deletion in position 15 of ITS 1 of *F. monophora*.

We derived the phylogenetic relatedness of our isolate with selected global isolates of *Fonsecaea spp.* fungus by using MEGA version 11.0.13 software (MEGA, https://www.megasoftware.net). We aligned the sequences by using multiple sequence comparison by log-expectation algorithm, followed by phylogenetic model determination. We implemented the maximum-likelihood method with the Kimura 2 parameter model with 1,000 bootstrap replicates (Figure 2).

**Figure 2:**
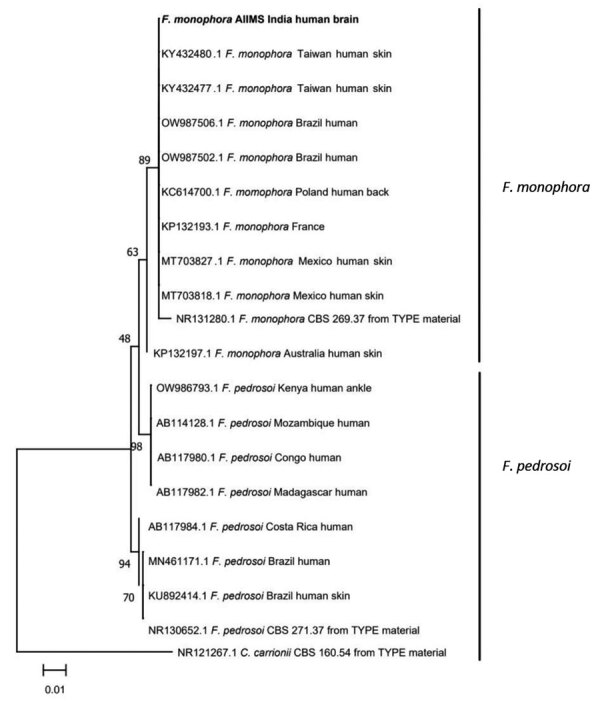
Phylogenetic relatedness of the isolate cultured from the brain biopsy sample from a 3-year-old patient in India with *Fonsecaea monophora* fungus infection (bold) compared with selected global isolates of *Fonsecaea* spp. Tree was derived by using the maximum-likelihood method with Kimura 2 parameter model and 1,000 bootstrap replicates implemented in MEGA 11.0.13 (MEGA, https://www.megasoftware.net).

*F. monophora* was first described as a separate fungal species by de Hoog et al. (*1*) in 2004. The next year, Surash et al. (*2*) published a case report of *F. monophora* CNS invasion. They also reported 2 previous cases of *F. monophora* CNS invasion identified retrospectively with sequencing (*3*,*4*). Since then, 8 other case reports of *F. monophora* CNS invasion have been published (*5*–*12*) (Appendix Table). Many cases of brain abscesses caused by *F. pedrosoi* fungus have been described in literature. Although most of them were reported before *F. monophora* was established as a species in 2004, some have been described more recently. Of note, none of the cases in the literature have had identifications confirmed by molecular methods. Both *F. monophora* and *F. pedrosoi* are causative agents of human chromoblastomycosis. Although *F. pedrosoi* is usually associated with chromoblastomycosis, *F. monophora* is considered a more general opportunist. *F. monophora* is also more neurotropic (*1*). Because *F. monophora* cannot be reliably differentiated from *F. pedrosoi* on the basis of phenotypic methods, some cases attributed to *F. pedrosoi* may have been caused by *F. monophora*. Molecular methods are essential for definitive identification. A review of brain abscess cases caused by *F. pedrosoi* was provided by Madhugiri et al. (*13*).

We have reviewed only cases in which CNS involvement was seen with *F. monophora*. In the cases we reviewed, *F. monophora* was limited to the CNS, except for 1 case where there was also involvement of the left foot (*5*). Headache was the most common symptom experienced and was accompanied by focal neurologic deficits and symptoms of increased intracranial pressure in some cases. Patient symptoms usually occurred for weeks before care was sought. Three cases were associated with multiple lesions in the CNS discovered by radiologic examination (*2*,*4*,*5*). In all the cases, final identification was confirmed by sequencing the ITS region from 18s rDNA extracted from culture growth.

Antifungal-susceptibility testing was done in 7 cases. Most results showed low MICs for the commonly used antifungals. Three of them reported MICs of >2 μg/mL for amphotericin B (*8*,*9*,*11*). Two reports described higher MICs for flucytosine (*9*,*10*). Those MICs are like results obtained previously from 25 clinical isolates of *F. monophora* (*14*). In a separate study on 10 clinical isolates of *F. monophora*, terbinafine and voriconazole were the drugs with the best in vitro activity, showing MICs <0.25 mg/L, whereas fluconazole, flucytosine, amphotericin B, caspofungin, and micafungin showed high MICs (*15*). Because voriconazole can penetrate the CNS well, it can be considered the drug of choice for CNS infections; amphotericin B monotherapy should be avoided. Terbinafine may also be considered in combination with voriconazole.

Six of the 11 patients we describe died. In 3 patients the immediate cause of death was not directly related to the fungal infection (*4*,*10*,*12*). Of the other 3 patients, surgical excision was not done in 2 (*3*,*9*). Of the 5 patients who survived, all were treated with surgical excision and various combinations of antifungals (*2*,*5*–*7*,*11*). All the patients improved greatly within weeks to months, and no recurrence of infection was reported after stopping antifungals. A combined approach with early surgical intervention and combination antifungals greatly improves outcome. Of note, serum (1,3)-β-D-glucan levels were elevated in 2 cases and decreased gradually with successful therapy (*6*,*11*). Thus (1,3)-β-D-glucan can be potentially used as a biomarker for follow-up.

## Conclusions

This case and review emphasizes the importance of molecular methods for the definitive identification of *F. monophora*, a cause of fungal brain abscess that is increasingly being reported. All the published cases to date have used sequencing for final identification. Targeting the ITS region of 18s rDNA, the universal fungal barcode, is usually sufficient, and no additional foci need to be sequenced. Although the implication of *F. monophora* identification for patient management is not clear, patient management may be changed in the future once more data are available.

Appendix Additional information about molecular identification of *Fonsecaea monophora*, novel agent of fungal brain abscess.
