# Supplementary material for: Molecular Identification of Fonsecaea monophora, Novel Agent of Fungal Brain Abscess
Source: Emerg Infect Dis. 2024 Jun;30(6):1232–5. doi: 10.3201/eid3006.240077 (PMC11138971; doi:10.3201/eid3006.240077)
Supplement: Appendix — Additional information about molecular identification of Fonsecaea monophora, novel agent of fungal brain abscess. [file 24-0077-Techapp-s1.pdf]

Article DOI: https://doi.org/10.3201/eid3006.240077

*EID cannot ensure accessibility for supplementary materials supplied by authors. Readers who have difficulty accessing supplementary content should contact the authors for assistance.*

# Molecular Identification of *Fonsecaea monophora*, Novel Agent of Fungal Brain Abscess

## Appendix

**Appendix Table.** *Fonsecaea monophora* cases reported in the literature

| Year/ Author | Age/Sex with Immune status and Exposure history | Symptoms | Radiology | KOH/ Direct Microscopy | Culture | Histopathology | Molecular | AFST | Therapy | Outcome/ Follow-up | Comment |
|---|---|---|---|---|---|---|---|---|---|---|---|
| 1954/ Lucasse (*1*) | 10/M | 4 mo of back pain refractory to quinine and penicillin; mild neck stiffness and occasional low-grade temperature; *Mansonella perstans* seen in blood smear | NA | small black filaments floating in clear CSF at autopsy | a black fungus thought at the time to represent *Cladosporium trichoides* | multiple small peripheral cerebral abscesses with a granulomatous response in autopsy specimen; some containing hyphal and spore-like forms | sequencing confirmed at CBS, Utrecht (CBS 100430) | NA | progressive neurologic deterioration despite empiric penicillin, streptomycin and antiparasitic therapy | death after 5 mo | Described as *Cladosporium trichoides* |
| 2003/ Nobrega (*2*) | 28/M with history of knife wound in right inguinal area with granuloma sixteen years back and noticed a visual defect in the left eye at that time; previous data for schistosomiasis and Chagas disease; more recently, manifested a pulmonary granulomatous lesion in the right lung with a single non-pigmented form of a fungus | severe bitemporal headache accompanied by fits of dizziness with nausea and vomiting, right ocular pain and loss of the temporal field of the right eye; right homonymous inferior quadrantopsy defect and a left homonymous hemianopsy; macrocytic anemia probably of nutritional origin | tumoral mass measuring 2 × 2 x 1.5 cm involving the right temporo-occipital area and another smaller apparently healed lesion at the left occipital lobe | elongated septate pigmented hyphae | velvety, dark olive gray colonies developing radial grooves and a central elevation when old; LPCB showing light brown septate hyphae ≈3 µm in diameter and straight conidiophores bearing frequently branched one-celled chains of conidia | granulomatous inflammation; golden brown round or oval forms 10–12 µm in diameter and septate hyphal forms 6–7 µm in diameter observed inside giant cells or isolated in necrotic areas (“chromoblastomycosis”); capillary hyperplasia and perivascular lymphocytic cuffing | sequencing confirmed at CBS, Utrecht | NA | IV amphotericin B started after pathology report; right temporal craniotomy and surgical enucleation after 20 d; discharged with oral itraconazole | initially responded to surgery and antifungals; expired few months after due to complications of the previous neuro-surgery; no residual fungal disease at autopsy | Described as *F. pedrosoi* |
| 2005/ Surash (*3*) | 53/M with diabetes, previous exposure to tropical fish and plants | 1 week history of right-sided weakness, slurred speech and constant | 3.6 × 2.3 cm ring-enhancing lesion in left frontal lobe with surrounding edema; | septate fungal hyphae on Gram stain | dematiaceous fungus after 1 week incubation provisionally | brown, septate, branching hyphae with occasional bulbous elements | sequencing confirmed at CBS, Utrecht (CBS 117238) | amphotericin B 0.5 µg/mL, itraconazole 0.03 µg/mL, | IV amp B for 1 week; IV voriconazole+flucytosine followed by switch to oral; surgical excision of frontal | stable and slowly improving | 1st described case of CNS *F. monophora* (3rd overall) |

| Year/ Author | Age/Sex with Immune status and Exposure history | Symptoms | Radiology | KOH/ Direct Microscopy | Culture | Histopathology | Molecular | AFST | Therapy | Outcome/ Follow-up | Comment |
|---|---|---|---|---|---|---|---|---|---|---|---|
| | | dull right-sided headache | new lesion within right supra-sellar cistern and adjacent anterior interhemispheric fissure after 4 mo of therapy; another lesion in left thalamus after 5 mo of therapy; good resolution after 12 mo of therapy | | identified as *Cladophialophora spp.* | | | voriconazole 0.06 µg/mL and posaconazole <0.015 µg/mL | lesion after 3 mo; oral voriconazole replaced with IV itraconazole after 5 mo followed by switch to oral; 21 mo of antifungals | | |
| 2007/ Takei (*4*) | 62/F post liver transplant 4 mo back | 5-mo history of progressive left upper extremity numbness as well as a 1-mo episode of increasing pain and swelling in the left ankle | three ring-enhancing lesions in cortex and subcortical white matter: 16 mm in right parietal lobe, 15 mm in left parietal lobe (solid), 5 mm in left occipital lobe; 11 × 13 mm enhancing subchondral geographic marrow abnormality within the left medial malleolus | NA | *.F monophora* grown from brain and tibial lesions | granulomatous inflammation with fungal organisms: groups of oval to elongated yeast-like cells, chains of budding cells (toruloid hyphae) and rare septate hyphae; with golden-brown cell walls and observed inside giant cells or isolated in necrotic areas; scattered muriform cells | done but not specified; final identification done at University of Texas, San Antonio | NA | surgical excision of right parietal lesion; oral voriconazole started on post-operative day 1 and continued; discharged after 9 d | no recurrence 1.5 y after discharge | 1st case involving multiple systems |
| 2010/ Koo (*5*) | 48/F post renal transplant 8 y back on prednisone, tacrolimus, mycophenolate mofetil and sulfamethoxazole for frequent urinary tract infection; previously two episodes of acute rejection, CMV reactivation; born and raised in Jamaica and emigrated to the U.S. 20 y back | progressively severe frontal headaches of two weeks duration, discomfort and stiffness of neck muscles and mild photophobia | irregular 3.1 × 3.4 cm ring-enhancing lesion in left frontal lobe with mass effect, an area of more solid-appearing enhancement | NA | velvety grayish-black mold showing septate hyphae with 4 types of conidial formations: *Rhinocladiella*-like, *Cladosporium*-like, *Phialophora*-like and thinner asterisk-like | granulomatous inflammation and numerous pigmented budding yeast-like and hyphal forms | sequencing confirmed at CBS, Utrecht (CBS 117542) | amphotericin B 0.25 µg/ml, voriconazole 0.125 µg/ml and posaconazole 0.03 µg/ml | surgical excision and empirical liposomal amphotericin B; voriconazole added after phaeohyphomycosis confirmed and both continued for 2 weeks; voriconazole monotherapy for 18 mo | no recurrence 4.5 y after discharge | (1→3) β-D-glucan (BG) levels >500 pg/ml with serial decrease |
| 2014/ Doymaz (*6*) | 71/F with chronic diabetes mellitus and hypertension | nausea, vomiting and headache | 3 × 4 × 3 cm lesion in left temporal lobe, 3 mm midline shift to the right side, vasogenic edema in the left fronto-temporo-parietal area | NA | dematiaceous fungus on SDA by 1st week; velvety in texture; LPCB suggestive of *Fonsecaea spp.*- up to three and | hyphal elements with thick septations, suppurative inflammatory changes, necrosis, foreign body giant cells and gliosis in peripheral parenchymal tissues | sequencing confirmed | NA | surgical excision and liposomal amphotericin B; replaced with IV voriconazole after 40 d for 3 weeks; discharged on day 60 on oral voriconazole | no recurrence 10 mo after discharge; healthy and stable | 4th described case of CNS *F. monophora* |

| Year/ Author | Age/Sex with Immune status and Exposure history | Symptoms | Radiology | KOH/ Direct Microscopy | Culture | Histopathology | Molecular | AFST | Therapy | Outcome/ Follow-up | Comment |
|---|---|---|---|---|---|---|---|---|---|---|---|
| | | | - preliminary diagnosis of glioblastoma multiforme | | very rarely small fourth conidia formed on the tips of the conidiophores | | | | | | |
| 2016/ Bagla (7) | 54/F PLHA diagnosed 9 mo back; on ART with CD4 count 42 cells/mm3 and undetectable viral load; history of hepatitis C without cirrhosis and late latent syphilis; IV drug abuse 20 y back; smoking and alcohol addiction | intermittent headache, vomiting and episodes of left arm twitching for 1 week; confused and incontinent of stool and urine; diminished muscle strength and deep tendon reflexes in left arm | 2 cm ring enhancing lesion with surrounding vasogenic edema in right parietal lobe | septate hyphae | dematiaceous mold | dematiaceous fungus in a background of granulomas with Langhans giant cells and central necrosis | sequencing confirmed at Associated Regional University Pathologists | amphotericin B 2 µg/mL, itraconazole 0.5 µg/mL, voriconazole 0.06 µg/mL and posaconazole 0.25 µg/mL | surgical excision on 7th day; started on IV voriconazole and liposomal amphotericin B on day 8; voriconazole changed to oral on day 12 | initially improved on antifungals (possible response to steroids); worsening mental status from day 16 and expired within days | 5th described case of CNS *F. monophora* |
| 2016/ Varghese (*8*) | 63/M with type 2 diabetes mellitus and decompensated chronic liver disease; past history of endoscopic esophageal variceal ligation | headache and progressive left-sided weakness for 3 weeks | 2 × 2 cm hypodense cystic lesion in right lentiform nucleus region with significant perilesional edema | branched, septate hyphae with light brown pigmentation | olivaceous to black in color with velvety appearance; pigmented septate hyphae and pale to olivaceous smooth-walled conidia on sympodially branched conidiophores on LPCB | NA | sequencing confirmed- EMBL accession no. LN626652 (ITS) and LN651287 (D1/D2) | E-test: amphotericin B 4 µg/ml, flucytosine >32 µg/ml, itraconazole 0.19 µg/ml, voriconazole 0.004 µg/ml, posaconazole 0.032 µg/ml, caspofungin 0.25 µg/ml and anidulafungin 0.38 µg/ml | started empirically on IV fluconazole; replaced with oral voriconazole after 1 week; repeat aspiration and amphotericin B added for neurologic deterioration after 2 weeks | expired due to raised intracranial tension despite antifungals; caregivers refused surgical intervention | 1st described case of CNS *F. monophora* from India |
| 2017/ Stokes (*9*) | 63/M South Sudanese immigrant with poorly controlled type 2 diabetes, liver cirrhosis due to chronic hepatitis B; recent travel to South Sudan for 1 y; admitted to an intensive care unit in Egypt for ≈1 mo with apparent malaria; progressive | headache and constitutional symptoms for weeks; febrile at his follow-up appointment 5 d later | CT head in the emergency was normal; 9 mm ring-enhancing lesion near the gray–white matter junction of the left parasagittal frontal lobe with a large amount of surrounding vasogenic edema on day 8; rapidly progressed to 20 mm on day 26 | branched, septate hyphae | dematiaceous mold that was identified as *F. monophora* | extensive tissue and blood vessel necrosis with septate branching hyphae | sequencing confirmed | flucytosine 4 µg/mL, amphotericin B 0.25 µg/mL, itraconazole 0.06 µg/mL, micafungin 1 µg/mL, caspofungin 0.25 µg/mL and voriconazole 0.25 µg/mL | ceftriaxone and metronidazole for empiric therapy; anti-tubercular therapy added after day 12; continued to deteriorate; liposomal amphotericin B started on day 38; IV voriconazole added on day 42 | right hemiplegia and worsening level of consciousness during admission; expired despite antifungals on day 64 from severe aspiration pneumonia | 7th described case of CNS *F. monophora* |

| Year/ Author | Age/Sex with Immune status and Exposure history | Symptoms | Radiology | KOH/ Direct Microscopy | Culture | Histopathology | Molecular | AFST | Therapy | Outcome/ Follow-up | Comment |
|---|---|---|---|---|---|---|---|---|---|---|---|
| | fatigue, a 20 kg weight loss, decreased appetite, diarrhea and diffuse abdominal pain since then; had previously received epidural injections, the last one >6 y earlier; had worked for an Alberta slaughterhouse | | | | | | | | | | |
| 2018/ Dobias (*10*) | 61/M from Moldova, living in the Czech Republic, who had worked as a locksmith on oil platforms in Turkmenistan, Kazakhstan, Sudan, and Iraq; myringotomy of both ear drums in 2010 | sudden motion disorder of the right limbs, dysarthria, and hypomimia; confusion since 3 mo; right cranial nerves palsy | expansive focus of 3. 1 × 5. 2 cm size around the left lateral ventricle of the brain and a pronounced peripheral edema | NA | dark-pigmented fungal colonies with macro- and micromorphological characteristics typical of the genera *Cladophialophora*/ *Fonsecaea* | dark-pigmented hyphae | sequencing confirmed directly from brain tissue and culture; GenBank accession numbers LT984660 and LT984661 | E-test: voriconazole 0.016 µg/mL, amphotericin B 4 µg/mL, itraconazole 0.25 µg/mL and posaconazole 0.016 µg/mL | surgical excision on day 5; liposomal amphotericin B; changed to voriconazole after AFST | improved significantly post-op, able to walk in 4 mo | (1→3) β-D-glucan (BG) levels 149 pg/ml with serial decrease |
| 2018/ Helbig (*11*) | 78/F with chronic obstructive pulmonary disease, presumably related to her work history as a baker | subacute right lower extremity paresis and facial nerve palsy | 18 × 13 mm left thalamic mass with surrounding edema; enlargement of the ring-enhancing mass (24 × 20 mm) at 1 week | branching septate hyphae morphologically suggestive of *Aspergillus* | black velvet-like colonies | septate branching hyphae with chronic and acute inflammation and necrosis | sequencing confirmed at Robert Koch Institute, Berlin | Voriconazole 0.125 µg/mL | IV dexamethasone started after imaging; radical open excision at 1 week; voriconazole added after AFST | initially improving with voriconazole; died 4 mo post-surgery due to pneumonia | 1st described case of CNS *F. monophora* from Germany |

## References


1. Lucasse C, Chardome J, Magis P. Cerebral mycosis from *Cladosporium trichoides* in a native of the Belgian Congo [in French]. Ann Soc Belg Med Trop (1920). 1954;34:475–8. **PMID: 13229098**

2. Nóbrega JP, Rosemberg S, Adami AM, Heins-Vaccari EM, Lacaz CS, de Brito T. *Fonsecaea pedrosoi* cerebral phaeohyphomycosis (“chromoblastomycosis”): first human culture-proven case reported in Brazil. Rev Inst Med Trop São Paulo. 2003;45:217–20. PubMed https://doi.org/10.1590/S0036-46652003000400008

3. Surash S, Tyagi A, De Hoog GS, Zeng JS, Barton RC, Hobson RP. Cerebral phaeohyphomycosis caused by *Fonsecaea monophora*. Med Mycol. 2005;43:465–72. PubMed https://doi.org/10.1080/13693780500220373

4. Takei H, Goodman JC, Powell SZ. Cerebral phaeohyphomycosis caused by *Ladophialophora bantiana* and *Fonsecaea monophora*: report of three cases. Clin Neuropathol. 2007;26:21–7. PubMed https://doi.org/10.5414/NPP26021

5. Koo S, Klompas M, Marty FM. *Fonsecaea monophora* cerebral phaeohyphomycosis: case report of successful surgical excision and voriconazole treatment and review. Med Mycol. 2010;48:769–74. PubMed https://doi.org/10.3109/13693780903471081

6. Doymaz MZ, Seyithanoglu MF, Hakyemez İ, Gultepe BS, Cevik S, Aslan T. A case of cerebral phaeohyphomycosis caused by *Fonsecaea monophora*, a neurotropic dematiaceous fungus, and a review of the literature. Mycoses. 2015;58:187–92. PubMed https://doi.org/10.1111/myc.12290

7. Bagla P, Loeffelholz M, Blanton LS. Cerebral phaeohyphomycosis by *Fonsecaea monophora*: report in a patient with AIDS and a ring enhancing lesion. Med Mycol Case Rep. 2016;12:4–7. PubMed https://doi.org/10.1016/j.mmcr.2016.06.002

8. Varghese P, Jalal MJA, Ahmad S, Khan Z, Johny M, Mahadevan P, et al. Cerebral phaeohyphomycosis caused by *Fonsecaea monophora*: first report from India. Int J Surg Med. 2016;2:44–9. https://doi.org/10.5455/ijsm.neurosurgery01

9. Stokes W, Fuller J, Meier-Stephenson V, Remington L, Meatherall BL. Case report of cerebral phaeohyphomycosis caused by *Fonsecaea monophora*. Off J Assoc Med Microbiol Infect Dis Canada. 2017;2:86–92. https://doi.org/10.3138/jammi.2.1.013

10. Dobias R, Filip M, Vragova K, Dolinska D, Zavodna P, Dujka A, et al. Successful surgical excision of cerebral abscess caused by *Fonsecaea monophora* in an immunocompetent patient and review of literature. Folia Microbiol (Praha). 2019;64:383–8. PubMed https://doi.org/10.1007/s12223-018-0661-9

11. Helbig S, Thuermer A, Dengl M, Krukowski P, de With K. Cerebral abscess by *Fonsecaea monophora*—the first case reported in Germany. Open Forum Infect Dis. 2018;5:ofy129. PubMed https://doi.org/10.1093/ofid/ofy129
